# Chikungunya Virus–Vector Interactions

**DOI:** 10.3390/v6114628

**Published:** 2014-11-24

**Authors:** Lark L. Coffey, Anna-Bella Failloux, Scott C. Weaver

**Affiliations:** 1 Center for Vectorborne Diseases, School of Veterinary Medicine, University of California, Davis, CA 95616, USA; E-Mail: lcoffey@ucdavis.edu; 2 Department of Pathology, Microbiology and Immunology, School of Veterinary Medicine, University of California, Davis, CA 95616, USA; 3 Department of Virology, Arboviruses and Insect Vectors, Institut Pasteur, 25-28 rue du Dr. Roux, 75724 Paris cedex 15, France; E-Mail: anna-bella.failloux@pasteur.fr; 4 Institute for Human Infections and Immunity, Center for Tropical Diseases and Department of Pathology, University of Texas Medical Branch, Galveston, TX 77555, USA

**Keywords:** chikungunya, mosquito, vector, transmission, adaptation

## Abstract

Chikungunya virus (CHIKV) is a mosquito-borne alphavirus that causes chikungunya fever, a severe, debilitating disease that often produces chronic arthralgia. Since 2004, CHIKV has emerged in Africa, Indian Ocean islands, Asia, Europe, and the Americas, causing millions of human infections. Central to understanding CHIKV emergence is knowledge of the natural ecology of transmission and vector infection dynamics. This review presents current understanding of CHIKV infection dynamics in mosquito vectors and its relationship to human disease emergence. The following topics are reviewed: CHIKV infection and vector life history traits including transmission cycles, genetic origins, distribution, emergence and spread, dispersal, vector competence, vector immunity and microbial interactions, and co-infection by CHIKV and other arboviruses. The genetics of vector susceptibility and host range changes, population heterogeneity and selection for the fittest viral genomes, dual host cycling and its impact on CHIKV adaptation, viral bottlenecks and intrahost diversity, and adaptive constraints on CHIKV evolution are also discussed. The potential for CHIKV re-emergence and expansion into new areas and prospects for prevention via vector control are also briefly reviewed.

## 1. Introduction

Emerging and re-emerging arthropod-borne viruses (arboviruses) represent a significant threat to human and veterinary health worldwide. Chikungunya virus (CHIKV), a mosquito-borne alphavirus that causes chikungunya fever (CHIK), a severe, debilitating and often chronic arthralgia, is a prime example; since it was first isolated in 1952, the virus has been detected as the etiologic agent of sporadic epidemics in Africa and Asia and, since 2004, has expanded its geographic range to circulate on Indian Ocean islands, and in Italy, France, and the Americas. CHIKV has also re-emerged in Southeast Asia since 2006, causing an estimated 1.3 million human cases [1]. CHIKV cycles in urban settings between humans and two mosquito species found in the U.S., suggesting a potential for endemic establishment there. As evidence of this, 11 autochthonous cases of CHIK were detected in south Florida as of November 2014 [2,3]. Emergence of arboviruses like CHIKV underscores the interconnectedness of humans with their environments, and highlights our vulnerabilities to new disease threats posed by spreading viruses. Understanding how arboviruses like CHIKV emerge is critical to predict and prevent or mitigate human disease. Central to understanding emergence is knowledge of the natural ecology of CHIKV transmission and the dynamics of vector infections. Here we discuss current knowledge of CHIKV infection dynamics in mosquito vectors and its relationship to human disease emergence.

## 2. Chikungunya Infection and Vector Life History Traits

### 2.1. Chikungunya Virus Transmission Cycles and Genetic Origins

Chikungunya virus is endemic in countries of Sub-Saharan Africa, India and Southeast Asia. The virus circulates in an enzootic cycle in Africa between forest-dwelling mosquitoes and non-human primates [4]. Phylogenetic studies reveal the existence of two major enzootic CHIKV lineages in Africa: Western, and East/Central/South African (ECSA) [5]. In Asia, where the first outbreak was reported in 1958 in Thailand, CHIKV has historically been maintained in an urban cycle transmitted to humans by the mosquito *Aedes* (*Stegomyia*)* aegypti* and, to a lesser extent,* Aedes* (*Stegomyia*)* albopictus* [6].

### 2.2. Chikungunya Distribution, Emergence and Spread

**Figure 1:**
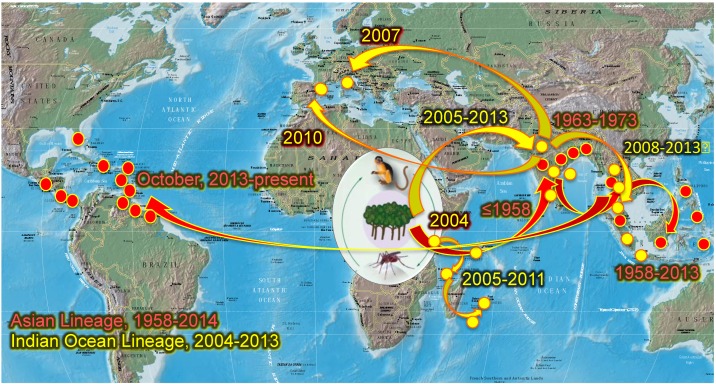
Geographic distribution and spread of Chikungunya virus (CHIKV) and its two urban vectors, *A. aegypti* and *A. albopictus*. Modified from reference [42], with permission.

In 2004, CHIKV belonging to the ECSA lineage emerged from Lamu and Mombasa in coastal Kenya [7] and spread to Comoros and later, to other islands of the Indian Ocean including La Réunion (Figure 1). There, the predominant vector *A. albopictus* transmitted preferentially a CHIKV variant with a single amino acid change from an alanine (A) to valine (V) at E1 envelope glycoprotein amino acid 226 of the ECSA Indian Ocean lineage (IOL) genotype [8]. The E1-226V variant was more efficiently transmitted by *A. albopictus* [9,10], with roughly 40-fold more efficient initial infection of midgut epithelial cells [9,10,11]. The selection of the E1-226V variant occurs at the initial infection of the midgut of *A. albopictus,* leading to a higher viral dissemination and transmission of the IOL genotype by this mosquito [12]. A series of four additional adaptive mutations in the E2 gene have also been incriminated more recently in enhancing transmission by *A. albopictus* [6,9,13]. During 2005–2006, the virus spread to neighboring Indian Ocean islands including Mayotte, Mauritius and Madagascar, where CHIKV E1-A226V was mainly transmitted by *A. albopictus* [12,14,15,16]. Subsequently, the CHIKV IOL was introduced to India [17,18] and the surrounding islands, Sri Lanka [19] and the Maldives. In Africa, the CHIKV E1-226V variant was also implicated outbreaks in Cameroon [20], Gabon [21,22] and Congo [23]. This variant also caused the first European CHIKV outbreak in Italy in 2007 [24]. Since 2008, IOL CHIKV strains were also imported into Southeast Asia: Malaysia [25], Singapore [26], Thailand [27], China [28], Cambodia [29] and Bhutan [30]. Remarkably, the E1-226V variant was found preferentially in rural areas where *A. albopictus* was more abundant than *A. aegypti*, and presumably was the primary vector [31,32]. In September 2010, autochthonous cases of CHIKV were reported in southeast of France [33,34], again with *A. albopictus* as the vector [34,35,36]. In Southeast France, this species appears to behave differently compared to its tropical counterpart, as it efficiently transmits the E1-226A IOL variant detected in local circulation [34,36]. *Aedes albopictus* has been found in in 18 French departments [34,35,37] as well as 19 other countries in Europe [38,39].

Prior to December 2013, CHIKV transmission was not documented in the Americas, despite numerous introductions and the presence of conditions that are apparently suitable for its establishment [6]. In October 2013, two laboratory-confirmed, autochthonous CHIKV cases were detected in the French territory of Saint Martin Island, in the Caribbean Sea. Surprisingly, the CHIKV strain isolated belonged to the Asian genotype [40] rather than the IOL that emerged in 2004 in the Indian Ocean Basin and Asia [6]. This St. Martin strain was phylogenetically close to CHIKV identified in Indonesia in 2007, China in 2012 and Philippines in 2013 [40], but more distant from the Asian genotype that circulated in New Caledonia [41]. The only vector implicated in St. Martin, where *A. albopictus* has not been established, was *A. aegypti*. Very rapidly, an epidemic was established in the island and subsequently, CHIKV progressively spread throughout most of the Caribbean, and into Central and South America where human populations are mostly naïve to CHIKV [42].

American populations of *A. aegypti* and *A. albopictus* are susceptible to both ECSA/IOL and Asian genotypes of CHIKV with higher susceptibility observed for *A. aegypti* and the Asian CHIKV genotype, or *A. albopictus* and the ECSA/IOL genotype [43]. As of November 2014, 11 locally acquired cases of CHIKV have been documented in Florida, USA [44]. These recent findings and the history of CHIKV movement suggest that it will continue to spread throughout the Americas and other tropical and subtropical regions of the world wherever mosquito vectors occur.

## 3. Vectors

### 3.1. Chikungunya Vector Distribution

CHIKV is an African virus that circulates enzootically in sylvatic cycle between arboreal, canopy-dwelling mosquitoes and non-human primates. Enzootic strains from the West African and ECSA lineages have been isolated from diverse mosquito species in Senegal, Ivory Coast, Central African Republic, Uganda and South Africa [4,45,46,47]. In Senegal, CHIKV has been detected in vectors of yellow fever virus: *Aedes* (*Diceromyia*)* furcifer*, *A.* (*Diceromyia*)* taylori*, *A.* (*Stegomyia*)* luteocephalus*, *A.* (*Stegomyia*)* africanus* and* A.* (*Stegomyia*)* neoafricanus* [47]. In South Africa, *A.* (*Diceromyia*)* cordellieri*, was also involved in CHIKV transmission [48]. The urbanization of CHIKV, with epidemics occurring in peridomestic settings following the emergence of enzootic strains, coincides with the involvement of anthropophilic mosquitoes: *A.* (*Stegomyia*)* aegypti* (L.) and *A.* (*Stegomyia*)* albopictus* (Skuse), favoring inter-human transmission. *Aedes aegypti*, first described by Linné in 1862, originated in African forests and is today present in most tropical countries [49]. In Africa, *A. aegypti* is present in two genetic forms: (i) the dark and sylvatic *A. aegypti formosus*, found in forested habitats and using treeholes as larval development sites; and (ii) the pale and domestic *A. aegypti aegypti*, which is widespread in the tropics and subtropics and uses artificial larval habitats mainly in urban environments [50,51,52]. *Aedes aegypti aegypti* originated from the forest-dwelling *formosus* form, which may have spread from tropical African forests to North Africa where it probably became domesticated under pressure to use artificial water storage containers as larval habitats [53]. These mosquitoes progressively differentiated into domesticated populations known as *A. aegypti aegypti*. Human trading activities later introduced this subspecies globally throughout the most of the tropics and subtropics: the New World via the African slave trade from the 15th to 19th centuries, Asia in the 18th and 19th centuries, and the Pacific islands with troop movements during World War II.

*Aedes aegypti* can exist sympatrically with* A. albopictus* and also often shares larval habitats [16,54,55,56]. *Aedes albopictus,* originally described by Skuse in Calcutta, India in 1894 originated in forests of Southeast Asia [57], but is now is commonly found in peri-urban, rural and forested areas on five continents [58]. *Aedes albopictus* has no particular ecological specialization, colonizing both temperate and tropical regions [59]. Two types of populations are described [60]: (1) temperate populations imported to the U.S. from Japan [61,62] and then from the U.S. to Europe [63,64], where they are now established in 20 European countries [58]; these temperate populations are characterized by diapausing, cold-resistant eggs [65]; and (2) tropical populations [60]. *Aedes albopictus* is a competent vector for at least 26 arboviruses [66] and is implicated occasionally in DENV (e.g., on the Seychelles islands [67]; and more frequently in CHIKV transmission (e.g., on La Réunion Island [68]; see Table 1 of this review as well). Since the 2007 outbreak of CHIKV in Italy [24], Europe is considered vulnerable for transmission of several “tropical” arboviruses, particularly in regions where *A. albopictus* is present [34,69,70].

### 3.2. Vector Dispersal and Genetics

*Aedes aegypti* and *A. albopictus* have both spread beyond their native ranges via commercial trade and dessication-resistant eggs [71]. *Aedes aegypti* largely replaced* A. albopictus* in Southeast Asian cities in the first half of the 20th century [65] while the introduction of *A. albopictus* into the Americas during the 1980s was associated with a decline in the abundance of *A. aegypti* in some regions such as occurred in the Americas in the 1980s [59,72,73] and is now ongoing in Central Africa [74,75] and on islands of the Indian Ocean region [16,76]. Long distance spread of CHIKV urban vectors beyond their natural flight ranges, typically a few hundred meters to a few kilometres [77], is usually achieved through transportation of immature stages (*i.e.*, larvae and eggs). From the 15th century onwards, successive waves of invasion by *A. aegypti* and, more recently, *A. albopictus*, have been facilitated by commercial transport.

Such mosquito invasions can be traced using molecular markers, which can now be developed from genome sequences. The *A. aegypti* genome sequence is complete [78] and genome annotations of *A. albopictus* are expected soon [79]. To assess mosquito phylogenetics, molecular markers used to define mosquito invasions have mainly been microsatellites (reviewed in [80]) and mtDNA [74,76,81,82,83,84]. Scenarios of invasions are more easily defined in island systems where mosquito populations have been geographically isolated with gene flow measurable through human displacements [85]. The involvement of human activities in reshuffling *A. albopictus* distributions has been demonstrated [86]. In Madagascar, *A. albopictus* dominant in the eastern coast and highland areas [87] are genetically distant from populations from South America and Southeast Asia [76]. This species continues to extend its geographic distribution in Madagascar, increasing its densities and progressively replacing *A. aegypti*, which is now present at residual levels in remote areas [76]. The decline in *A. aegypti* was also detected in the neighboring island of La Réunion [16,88].

The situation with these mosquitoes in Central Africa is slightly different. The invasion of *A. albopictus* occurred via several successive waves within a very short time frame. In the Central African Republic (CAR) *A. albopictus* first detected in 2008 [89], probably introduced from Cameroon where the species has been established since 2000 [90]. *Aedes albopictus* in Cameroon derives from multiple introductions from tropical sources that still need to be identified [74]. The polymorphism of mtDNA markers there is low, suggesting that *A. albopictus* were periodically introduced and that these events coincided with a decrease in *A. aegypti* densities [75].

### 3.3. Vector Competence

**Figure 2:**
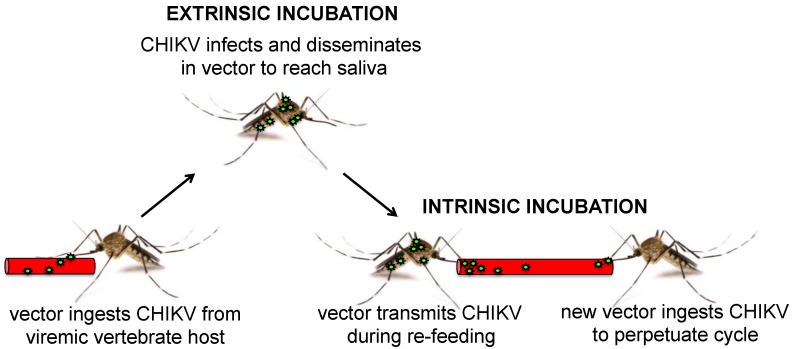
Process of infection and transmission of CHIKV by mosquito vectors. Vector mosquitoes ingest CHIKV from viremic vertebrate hosts during bloodfeeding. During the extrinsic incubation period that occurs in the vector, CHIKV infects the midgut and disseminates through the body cavity to infect salivary glands for secretion into saliva. Re-feeding vectors transmit CHIKV to vertebrate hosts by expectorating virus in saliva. New vectors perpetuate the CHIKV cycle by ingesting virus during intrinsic incubation, a period of viremia in the vertebrate host. Vector competence assays test infection, dissemination, and transmission of CHIKV after extrinsic incubation.

Vector competence, the ability of an arbovirus vector to acquire a pathogen and successfully transmit it to another susceptible host, is a complex process influenced by external factors including temperature, the availability of vertebrate hosts, vector population density and predation, as well as internal factors including mosquito survival and virus replication. For CHIKV, horizontal transmission through saliva that is injected when a mosquito probes or feeds on blood is the most common mechanism of transmission (Figure 2), although vertical transmission via infected eggs may also occur at a low rate [91]. Vector competence is typically evaluated experimentally using static laboratory-based methods, where assessment of virus replication is the primary endpoint. For CHIKV, mosquitoes from endemic locations are usually presented artificial bloodmeals loaded with known titers of virus from sympatric settings. Mosquitoes that imbibe infectious bloodmeals are incubated at a constant temperature for at least seven days and then killed to assess replication. The fractions of mosquitoes that become infected, develop infections that have disseminated from the initial infection site in the midgut into the hemocoel, and expectorate CHIKV in saliva are then measured to extrapolate vector competence for the population.

Table 1 summarizes experimental CHIKV vector competence estimates in arthropods from around the world. Most studies have focused on the two primary urban vectors, *A. aegypti* and *A. albopictus*, although other mosquitoes and one tick species have also been tested. Most studies have used relatively high bloodmeal titers exceeding 7 log_10_ plaque forming units (PFU)/mL (but that are still below peaks in infected humans [92]) and mosquitoes were typically sampled more than seven days post-feed, experimental designs that maximize the chances of CHIKV infection and transmission. Infection, dissemination, and transmission rates of *A. aegypti* and *A.* *albopictus* vary according to the geographic sources of mosquitoes tested. At bloodmeal titers of ≥7 log_10_ plaque-forming units (PFU)/mL, at least 80% of *A. aegypti* from all locations develop disseminated infections. More than half of *A. albopictus* that ingest bloodmeals ≥7 log_10_ PFU/mL also became infected or develop disseminated infections, with several exceptions. Infection and dissemination rates in *A. albopictus* from the US are dose-dependent and increase with the titer of the ingested bloodmeal [9,93]; dose response studies have not been published for mosquitoes from other regions. *Aedes albopictus* also exhibits strain-specific susceptibility; fewer mosquitoes from La Réunion Island, Mayotte, and colonized mosquitoes originally from Texas infected in parallel with a CHIKV isolate from early in the 2005–6 La Réunion Island outbreak developed disseminated infections than mosquitoes infected with strains isolated later in the outbreak (subsequent studies discussed below revealed that A to V substitution in amino acid 226 of the E1 protein (E1-A226V) mediates much of this differential infectivity [9,10,94]). In an attempt to exhaustively present CHIKV vector competence data, Table 1 shows all published data; however, comparing CHIKV vector competence over time in mosquitoes sampled from the same areas entails comparing data from experiments with unmatched bloodmeal titers. Differences could result from varied ingested doses rather than real differences in susceptibility to infection, dissemination and transmission. Despite this, studies from many locations worldwide show that *A. aegypti* and *A. albopictus* are generally highly susceptible to infection, dissemination and transmission of CHIKV. Alternate vector species, also represented in Table 1, may also serve as efficient CHIKV vectors in specific geographic settings; e.g., *Opifex fuscus* mosquitoes from New Zealand are highly competent at transmitting CHIKV from India.

### 3.4. Chikungunya Virus Vector Immunity and Microbial Interactions

While most arboviruses induce significant morbidity and/or mortality in some vertebrate hosts, infections of arthropod vectors are generally considered non-pathogenic. However, interactions between the replicating virus and the mosquito immune defense system produce an outcome that may influence subsequent viral dissemination or superinfection by other viruses (reviewed in [95]). Considerable progress has been achieved in understanding the innate defenses of the mosquito against arboviruses. The virus most intensively studied, DENV, triggers several immune pathways in *A. aegypti*: the Toll [96,97], JAK-STAT [98], and Imd/JNK pathways [99]. However, RNA interference (RNAi) appears to be the most significant innate antiviral immune response in mosquitoes [99,100,101,102,103,104,105]. So far, three major types of small RNA molecules have been identified in mosquito vectors: small interfering RNA (siRNA), microRNA (miRNA) and PIWI-interacting RNA (piRNA). These molecules have distinct roles in different cellular processes and virus-host interactions [106]. The antiviral siRNA pathway is triggered when long double-stranded RNA molecules are produced from secondary RNA structures and/or viral replication intermediates during infection of the vector. This leads to an activation of the RNA degradation machinery to the target viral RNA [100]. These pathways may act in a virus-specific manner.

Little is known about immune responses induced by CHIKV infection of mosquitoes. Viral replication is controlled *via* the exogenous RNAi pathway in mosquitoes [107]. The protein Ago-2 plays an important role in the antiviral RNAi response to CHIKV, similar to its role for other alphaviruses including, Sindbis (SINV) [102], Semliki Forest [108] and o’nyong-nyong viruses [101]. Viral replication under RNAi control may limit potential pathologic effects to favor mosquito survival [94].

The repeated use of insecticides to target mosquitoes as a means to control vector-borne diseases has found its limits due to the development of resistance [109], and alternative approaches are urgently needed. Recent strategies for controlling viral transmission have come from research on RNAi, such as the development of *A. aegypti* mosquitoes expressing small RNAs that render them resistant to viral infection; this approach is a promising mechanism for suppressing virus replication in mosquitoes [110,111,112]. Depending on the objectives, mosquito populations can also be reduced in density using various strategies including the Sterile Insect Technique (SIT), a SIT-like system called Release of Insects carrying a Dominant Lethal (RIDL) [113], or replaced by transmission-refractory mosquitoes [114]. For this latter strategy, *Aedes* mosquitoes have been successfully transformed using transposon vectors [115] or infections with the intracellular bacterium *Wolbachia* [116]. However, a system is required to spread the refractory genes into mosquito populations as well as to maintain the expression of the refractory phenotype through generations. Several gene-driver systems have been proposed [117] including the Medea system, which shows promise by coupling genes conferring disease refractoriness with a genetic mechanism for driving them through wild populations [118].

In addition to their ability to shorten mosquito life span [119], certain strains of *Wolbachia* are also able to reduce arboviral transmission [120,121]. *Wolbachia* induce various distortions of host reproduction via a form of sterility known as cytoplasmic incompatibility (CI), thereby promoting its spread into populations [122]. Therefore, certain strains of *Wolbachia* provide the double role of a gene driver system and carrier of a refractory phenotype. While the mosquito *A. aegypti* is free of natural endosymbiotic bacteria *Wolbachia*, some *A. albopictus* populations are naturally super-infected with two *Wolbachia* strains, *w*AlbA and *w*AlbB. These *Wolbachia,* present in mosquito midguts and salivary glands [123], do not affect CHIKV replication [124]. In contrast, *Wolbachia* are able to limit DENV in *A. albopictus* [125] but cannot completely block transmission [126]. Moreover, *A. albopictus* transfected with a heterologous *Wolbachia* isolated from the fruit fly *Drosophila melanogaster* (*w*Mel) inhibit the transmission of CHIKV [127] as in *A. aegypti* [121,128]. Several mechanisms have been proposed to explain the molecular basis of the pathogen-blocking *Wolbachia* infection phenotype: upregulation of immune genes, production of reactive oxygen species, or competition for a limited resource such as cholesterol [129,130,131].

In their digestive tracts, wild populations of *A. albopictus* and *A. aegypti* have been shown to house Proteobacteria and Firmicutes, including the genera *Acinetobacter*, *Asaia*, *Delftia*, *Pseudomonas*, *Wolbachia* and *Bacillus* as well as members of the family Enterobacteriaceae [132]. A higher diversity of bacteria can be occasionally found in wild *A. albopictus*; 27 genera of cultivable bacteria have been detected in this species from Madagascar, with Pantea bacterium as the most prevalent [133]. Other bacterial members of the Alpha- and Gammaproteobacteria phyla, as well as Bacteroidetes, respond to CHIKV infection [132]. The abundance of bacteria belonging to the Enterobacteriaceae family increases with CHIKV infection [132], suggesting that cooperation or competition occurs within the host. Microbiota (including *Wolbachia*) endogenous to some mosquito species pre-activate the expression of basal genes in the immune response (*i.e.*, immune priming), allowing the vector to be prepared for infection by pathogens. In *A. aegypti* infected with DENV, the arthropod microbiota elicit basal immune responses that act against the virus, and this response reduces the density of the microbial load in the mosquito midgut [134]. Antiviral activities can be induced by secreting antiviral compounds; *Serratia odorifera* in *A. aegypti* enhances susceptibility to CHIKV by interaction of its P40 protein with the mitochondrial protein porin present on the midgut brush border membrane of the mosquito midgut, thereby downregulating mosquito immune responses [135].

### 3.5. Vector co-Infection by Chikungunya and Other Arboviruses

Mosquitoes may feed several times during their lifespan and can ingest genetically distinct variants of the same virus species or even viruses from different families. Once a virus has infected the mosquito salivary glands, the mosquito becomes competent for transmission to the next vertebrate host, usually for the remainder of its life [136,137]. The mosquito can therefore host a collection of diverse viruses, playing a role in selecting genotypes involved in epidemics [138].

Because CHIKV circulates in DENV-endemic regions where the anthropophilic mosquitoes *A. albopictus* and *A. aegypti* can transmit both viruses, reports of co-infection in humans are increasing. Since the first observation in 1964 in South India [139], co-infections have been reported since the 2004 re-emergence: Sri-Lanka [140], India [141,142,143], Malaysia [144], Gabon [145], Madagascar [14], Singapore [146], and Angola [147]. The increasing number of reports of co-infections seems to coincide with introductions of *A. albopictus* [145]. In Gabon, between 2007 and 2010, 0.9% of 4287 acutely febrile patients were positive for both CHIKV and DENV, and more unexpectedly, co-infected *A. albopictus* were also collected [148]. Patients were possibly co-infected with the two viruses through the bite of a single mosquito, as has been demonstrated experimentally [149]. Considering the nearly worldwide circulation of DENV and CHIKV, co-infections may become more frequent. Concurrent infections may make diagnosis more challenging and could also result in different disease syndromes. Concurrent epidemics of yellow fever virus and CHIKV were also reported in Africa [150,151,152] and patients infected with the two viruses were suspected [153]. In the same way, Zika virus (ZIKV), typically transmitted in urban settings by *A. aegypti*, is also transmitted by *A. albopictus*, whose expanding distribution may favor the cocirculation of CHIKV and ZIKV [154].

## 4. Chikungunya Virus

### 4.1. Genetics of Vector Susceptibility and Host Range Changes

As described above, CHIKV circulates in two distinct transmission cycles: (1) enzootic transmission among nonhuman primates and perhaps other vertebrates by arboreal *Aedes* spp. mosquitoes in sub-Saharan African sylvatic foci, and; (2) urban transmission among humans by *A. aegypti* and/or *A. albopictus*. Like interactions between other arboviruses and their arthropod vectors, susceptibility to CHIKV infection of mosquitoes and their ability to transmit depend on the genetics of both, and differences in these properties can affect circulation and human exposure as described above. Enzootic vector-CHIKV interactions have received little experimental study [47]. Although population-based differences in urban vector competence may occur, these have not been addressed in enough detail to be conclusive or to begin to assess genetic components of vector susceptibility. Following the detection of the *A. albopictus*-adaptive A226V substitution in the E1 envelope glycoprotein during the 2005–2006 Réunion Island epidemic [8,9,10], the impact of CHIKV genetics on urban vector infection and transmission has received considerable study. Vector-adaptive evolution had been previously described for DENV [155,156] and the alphavirus Venezuelan equine encephalitis virus (VEEV) [157], the latter also involving a substitution in an envelope glycoprotein, in this case E2. Surprisingly, neither vector-adaptive alphavirus mutations has been shown to have much effect on infection of the previous or donor vector, *A. aegypti* in the case of CHIKV [9,12] and *Culex* (*Melanoconion*) *taeniopus* in the case of VEEV [158], challenging the hypothesis that most host-specific viral adaptations will have tradeoffs for fitness in other hosts. Further phylogenetic/reverse genetic studies of IOL CHIKV revealed an unprecedented series of four second-step, *A. albopictus*-adaptive mutations, each involving E2 substitutions, and one relying also on a synergistic effect of an E3 substitution [13,159]. Each of these mutations enhances initial infection of the mosquito midgut and has little or no effect on infection of *A. aegypti*. Furthermore, at least one combination of these independently acquired second-step mutations leads to an *A. albopictus* infection phenotype more efficient than that of any natural CHIKV strain studied to date, suggesting further vector-adaptive evolution and even more efficient circulation in regions where this mosquito is abundant. Structural modelling of these *A. albopictus*-adaptive envelope glycoprotein substitutions suggests that they alter the entry process in endosomes by affecting conformational changes required for E1 fusion with endosomes rather than directly affecting receptor binding [12,13].

### 4.2. Population Heterogeneity & Selection for Fittest Genomes

Alphaviruses like CHIKV exist as heterogeneous populations of viral RNAs called mutant swarms that arise from frequent nucleotide misincorporation during replication due to the inability of the viral RNA-dependent RNA polymerase (RdRp) to error correct. Given a ≈12 kb CHIKV genome and a mutation frequency of ≈10^−4^, each new RNA genome possesses one mutation on average. By comparison, double-stranded DNA virus mutation rates are several log_10_ lower [160]. Most mutant genomes are detrimental and removed from the swarm via purifying negative selection. By contrast, positive selection of a fit phenotype results in increased abundance of a genotype. Therefore, a dynamic mutation-selection balance determines the size and genetic diversity of a mutant swarm. Genetic diversity renders a population less prone to the consequences of negative selection pressures that target certain genotypes and renders a population more likely to contain variants with potential phenotypic advantages; these features can enhance plasticity and adaptability. A high fidelity CHIKV variant with a point mutation at amino acid position 483, a fidelity-determining locus in the RdRp, that was discovered experimentally after treatment with chemical mutagens, generates 30% fewer mutants than wild-type (wt) virus and is less fit in vectors and a mouse model [161,162]. This reduced fitness may result from the less mutated population containing fewer genotypes that can resist evolutionary pressures including negative selection or population bottlenecks. Complementary studies with low fidelity variants of CHIKV generated by mutagenizing the amino acid at 483 produce more errors than wt variants and are also attenuated in mosquito cells and mice [138]. Together these studies indicate that CHIKV maintains an intermediate mutation frequency to avoid detriments to fitness resulting from populations with too few or too many mutant genomes.

### 4.3. Dual Host Cycling & Chikungunya Virus Adaptation

Strong purifying selection of alphaviruses that cycle between mosquitoes and vertebrates results in less genetic variation than predicted by their high mutation rates [138,163,164,165]. Work with other alphaviruses including eastern equine encephalitis virus (EEEV), SINV, and VEEV shows that alternating between hosts poses conflicting challenges to replication that can limit adaptation to either host alone by imposing fitness costs where adaptations are antagonistic ([166] reviewed in [167]). When one host is artificially removed via experimental serial passage, the limitations of these trade-offs are evident; viruses serially passaged in a single host are more adaptable. CHIKV serially passaged in vertebrate or mosquito cells exhibits higher fitness when passaged in novel cell types and also showed enhanced neutralization escape and antiviral compound resistance. These changes are accompanied by increased genetic diversity. In contrast, alternating CHIKV passage between cell types restricts fitness and increases diversity, suggesting that only mutations beneficial or neutral in both host cells are maintained and that these variants retain fitness in alternating cycling [168].

### 4.4. Viral Bottlenecks and Intrahost Diversity

The ability to circumvent bottlenecks within and between dynamic environments including switching between vector and vertebrate hosts impacts CHIKV evolution and is important for understanding changing population dynamics that ultimately cause human disease. Bottlenecks that reduce arbovirus population size can influence viral fitness by restricting phenotypic plasticity that stems from having genetic diversity. The evolutionary theory Muller’s ratchet asserts that asexual organisms with high mutation rates and small population sizes irreversibly accumulate deleterious mutations unless compensatory mutations restore mutation-free genomes to the population [169]. Studies with EEEV [170] validate this concept; fitness decreases after serial bottleneck passages can be rescued by subsequent large population passages, albeit with much replication needed to overcome the ratchet [171]. Anatomical barriers to productive alphavirus transmission by mosquito vectors are relatively well defined, although only one study has addressed CHIKV directly. As a first step, alphaviruses in a bloodmeal ingested by a mosquito must infect the midgut epithelium. Some VEEV studies suggest that only “portal” cells in the midgut epithelium are susceptible to infection [172]; other experiments show uniform susceptibility [173], possibly reflecting a longer virus-vector relationship for the latter. Secondary impediments to dissemination result in failure of the virus to escape from the midgut epithelium, infect salivary glands, and escape from salivary gland cells into saliva for transmission to vertebrates. The number of barcoded VEEV variants that successfully traverse these barriers is reduced at midgut escape and salivary gland infection compared to the ingested bloodmeal, and smaller initial bloodmeal populations are more prone to reductions in variant diversity [174], suggesting strong genetic bottlenecks that reduce diversity coincident with population size changes. These observed changes in genetic diversity of the mutant swarm in mosquitoes contrast with observations from other mosquito-borne arboviruses isolated from nature [175], as well as experimental* in vivo* passaging studies with VEEV that show the maintenance of genetic and phenotypic stability of the consensus (average sequence) [176]. The disparity in results between these studies may be explained by the re-establishment of genetic diversity after bottlenecks via subsequent replication. Studies with CHIKV support this idea; although population diversity in the midgut and salivary glands is reduced compared to the bloodmeal input or midgut population, respectively, it recovers downstream of each barrier and the consensus sequence remains unchanged [138].

Experimental studies showing that alternating hosts impose constraints on arboviruses including CHIKV indicate that inefficient transmission probably interrupts natural CHIKV cycling. However, the degree of natural extinction in vertebrate or vector hosts has not been directly addressed. Extinction in an individual mosquito-vertebrate-mosquito lineage is likely because most mosquitoes do not survive long enough in nature to feed more than once. The maintenance of consensus genetic stability in nature in the presence of intense circulation may be due to the extinction of most individual lineages, possibly via vector bottlenecks in individual mosquitoes. However, these extinctions must not be widespread enough; otherwise CHIKV cycling would be interrupted. No studies published on CHIKV or other alphaviruses have examined mutant swarms in naturally infected mosquitoes, humans, or other vertebrate hosts. Artificially generated, barcoded VEEV variants in mosquitoes that transmitted to laboratory mice were also observed in the brains of animals [174], suggesting that mosquito-to-vertebrate transmission of alphaviruses does not present a major bottleneck (although few (*n* = 3) infected mice were sampled). Virus doses expectorated by mosquitoes vary greatly, and are probably overestimated by standard laboratory salivation assays where infected mosquitoes eject more virus into tubes than* in vivo* [177]. Therefore, variance in transmitted doses, especially when few particles are transmitted, likely impacts the outcome of vertebrate infection and maintenance of alphavirus cycling in natural foci. Interruptions in seasonal transmission may represent another significant bottleneck imposed on CHIKV, especially if the virus is introduced into temperate climates [178] where vector survival and competence would decrease in colder temperatures [179]; however this phenomenon is complex [180] and has not been studied extensively.

### 4.5. Adaptive Constraints on Chikungunya Virus Evolution

Although the 2004 IOL CHIK emergence underscores the adaptive potential of RNA viruses including most arboviruses, it also provides examples of constraints on adaptive evolution that remain difficult to predict. Although the E1-A226V substitution was selected convergently after IOL strains reached locations with abundant *A. albopictus*, it surprisingly was not found in any CHIKV strains of the Asian lineage despite their circulation in regions of Asia native to this vector for more than 60 years [5]. This lack of adaptation to *A. albopictus* in Asia resulted from an epistatic interaction with E1 residue 98; Asian CHIKV strains, which have a threonine residue at position 98, show no increase in *A. albopictus* infectivity when the E1-A226V substitution is engineered into a cDNA clone derived from the Asian strain, while ECSA and IOL strains with 98A show a dramatic increase in infection. The lack of sequenced enzootic CHIKV strains with 98T suggests that this residue, which by itself has no detectable effect on infection of urban vectors or models for human infection, resulted from a founder effect when CHIKV was introduced into Asia sometime before 1958 [181].

Although variation among Asian strain CHIKV infection of *A. aegypti* has received little study, no evidence has been produced to support adaptive evolution in Asia since the 1950s. The above evidence indicating an adaptive constraint on Asian strains, including those now circulating in the Americas, for enhanced transmission by *A. albopictus* suggests that *A. aegypti* will remain the principal vectors as spread into American regions inhabited by both vectors continues [13]. However, additional studies of the Asian lineage and its potential to increase transmissibility by either mosquito are needed.

## 5. Future Prospects

### 5.1. Potential for Re-Emergence and Expansion into New Areas

Due to the immunologically almost completely naïve status of human populations and the widespread and abundant *A. aegypti* nearly throughout the western hemisphere tropics, CHIKV is expected to continue to spread and ultimately recapitulate the distribution of DENV in the Americas. The lack of historical evidence for a major role of *A. albopictus* in transmission of Asian lineage CHIKV strains and the adaptive constraint described above suggest that temperate American regions inhabited by *A. albopictus* but not *A. aegypti* may not be at as high a level of risk as regions where IOL strains are circulating. However, IOL strains continue to circulate in Asia and have a history of many importations into the Americas [182], so their risk of introduction into the Americas continues. Should both Asian and IOL strains cocirculate, as they have in Southeast Asia since 2007, even more geographic regions of the Americas could be at risk for CHIK.

### 5.2. Prospects for Prevention via Vector Control

The poor history of DENV control since the 1970s suggests that mitigation of CHIKV transmission via vector control will be highly challenging. Due to its tight association with artificial larval habitats, endophily of adult females, and daytime biting patterns, control of *A. aegypti* and DENV transmission is difficult, and *A. albopictus* presents similar challenges. The development of resistance to commonly used insecticides further complicates the control of these vectors. Although some novel strategies for *A. aegypti* control discussed above such as RIDL offer promise, they remain in the early stages of field testing [183]. Interim measures including the application of persistent insecticides to the interior of houses may be needed in some situations to reduce CHIKV and DENV transmission.

The long-term prospects for CHIKV maintenance in the endemic, urban cycle are not entirely clear. Following introduction of the Asian lineage into South and Southeast Asia during the 1950s or earlier, it became extinct in India after 1973 for unknown reasons, but continues to circulate in Southeast Asia today [167,184]. The only major human immune cross-reactivity known to affect CHIKV to a major extent is that from Ross River and Mayaro viruses, which like CHIKV are members of the Semliki Forest complex of alphaviruses. Ross River virus only occurs in Australia and Indonesia, and Mayaro virus in South America where seroprevalence is generally low in urban settings. These data suggest that CHIKV will continue to circulate indefinitely in Asia and probably in the Americas as well.

**Table 1:** Chikungunya virus vector competence in arthropods. Infection was ascertained by detection of virus in bodies of bloodfed mosquitoes; dissemination was determined by detection of virus in legs or heads. Transmission was verified by detection of virus in saliva or by infection of vertebrates after re-feeding. CHIKV bloodmeal titers are expressed in in log_10_ cell culture infectious dose_50_/mosquito, plaque forming units/mL, or suckling mouse infectious culture lethal dose_50_/mL. Mosquitoes were held at 28 °C during the incubation period, except where noted: * indicates incubation at 24 °C and ** denotes incubation at 16 °C. For simplification, cohorts of the same species that fed on the same strain at similar bloodmeal titers are represented as one value and bloodmeal titers are shown as ranges.

Mosquito Species	Source	Generation Number	Source of CHIKV, Isolation Year	Strain Name	Bloodmeal Titer	Incubation Period (Days)	% Infected (n)	% Disseminated (n)	% Transmitted (n)	Reference
*Aedes aegypti*	Queensland, Australia	1	patient in Melbourne ex. Mauritius, March 2006	not stated	4	14-15	92 (23/25)	92 (23/25)	64 (16/25)	van den Hurk* et al.*, 2010 [128]
	New Caledonia	1	patient in New Caledonia ex. Indonesia, February 2011	NC 2011-568	7	14	n.d.	n.d.	27 (10/37)	Dupont-Rouzeyrol* et al.*, 2012 [41]
			patient in Reunion Island, 2006	6.21			n.d.	n.d.	75 (27/36)	
	Mayotte	3	patient in Reunion Island, 2005 (E1226V)	6.21	7.5	14	n.d.	87 (54/62)	n.d.	Martin* et al.*, 2010 [94]
			patient in Reunion Island, 2005 (E1226A)	6.115			n.d.	78 (43/55)	n.d.	
	Cameroon	1	patient in Reunion Island, 2005 (E1226V)	06.21	7	14	n.d.	89 (333/376)	n.d.	Paupy* et al.*, 2010 [185]
			patient in Reunion Island, 2005 (E1226A)	06.115			n.d.	97 (36/37)	n.d.	Vazeille* et al.*, 2007 [10]
			patient in Reunion Island, 2005 (E1226V)	06.21			n.d	65 (70/107)	n.d.	
			patient in Mayotte, 2006	06.111			n.d.	82 (68/82)	n.d.	
			patient in Democratic Republic of Congo, 2000	06.117			n.d	84 (56/66)	n.d.	
	Dakar, Senegal	4	patient in Thailand, 1962	15561	4.2–4.6	7	2 (1/45)	0 (0/45)	n.d.	Turell* et al.*, 1992 [186]
	Lagos, Nigeria	colony	patient in Calcutta, India, 1963	63-266	8	14	10 (2/20)	n.d.	50 (1/2)	Shah* et al.*, 1964 [187]
	French West Indies	1-2	patient in Reunion Island, 2006	06.21	7.5	14	98 (900/918)	n.d.	n.d.	Girod* et al.*, 2011 [188]
			patient in Reunion Island, 2006	06.21	6	7	47 (301/634)	n.d.	n.d.	
	Trinidad, West Indies	colony	patient in Calcutta, India, 1963	63-266	8	14	42 (3/7)	n.d.	100 (1/1)	Shah* et al.*, 1964
*Aedes aegypti*	Guadeloupe	1	patient in Reunion Island, 2005 (E1226V)	6.21	7.5	14	n.d.	96 (346/358)	n.d.	Girod* et al.*, 2011
	Martinique	1	patient in Reunion Island, 2005 (E1226V)	6.21	7.5	14	n.d.	98 (285/290)	n.d.	Girod* et al.*, 2011
	French Guyana	1	patient in Reunion Island, 2005 (E1226V)	6.21	7.5	14	n.d.	99 (269/270)	n.d.	Girod* et al.*, 2011
	Florida, USA	1	patient in France ex. Reunion Island, 2006	LR2006-OPY1	6.1	6	58 (15/26)	73 (11/15)	n.d.	Pesko* et al.*, 2009 [93]
			patients in Reunion Island, 2005	06.21 and/or 06.115	7.5	10	n.d.	100 (48/48)	n.d.	Vega-Rua* et al.*, 2014 [43]
	Louisiana, USA	4-5	patient in Thailand, 1962	15561	4.2–4.6	7	5 (3/60)	2 (1/60)	n.d.	Turell* et al.*, 1992
	Indiana, USA	7	patient in Thailand, 1962	15561	4.2–4.6	7	6 (2/35)	6 (2/35)	n.d.	Turell* et al.*, 1992
	Puerto Rico, USA	3	patient in Thailand, 1962	15561	4.2–4.6	7	55 (9/55)	11 (6/55)	n.d.	Turell* et al.*, 1992
		5	patient in Thailand, 1962	15561	5.3	7	33 (10/30)	10 (3/30)	n.d.	
	Mexico	1	patients in Reunion Island, 2005	06.21 and/or 06.115	7.5	10	n.d.	97 (58/60)	n.d.	Vega-Rua* et al.*, 2014
	Panama	1	patients in Reunion Island, 2005	06.21 and/or 06.115	7.5	10	n.d.	97 (58/60)	n.d.	Vega-Rua* et al.*, 2014
			patient in New Caledonia, 2011	NC/2011-568			n.d.	100 (30/30)	n.d.	
	Venezuela	1	patients in Reunion Island, 2005	06.21 and/or 06.115	7.5	10	n.d.	100 (51/51)	n.d.	Vega-Rua* et al.*, 2014
	Peru	1	patients in Reunion Island, 2005	06.21 and/or 06.115	7.5	10	n.d.	100 (89/89)	n.d.	Vega-Rua* et al.*, 2014
	Brazil	1	patients in Reunion Island, 2005	06.21 and/or 06.115	7.5	10	n.d.	98 (128/130)	n.d.	Vega-Rua* et al.*, 2014
			patient in New Caledonia, 2011	NC/2011-568			n.d.	95 (57/60)	n.d.	Vega-Rua* et al.*, 2014
	Bolivia	1	patients in Reunion Island, 2005	06.21 and/or 06.115	7.5	10	n.d.	100 (60/60)	n.d.	Vega-Rua* et al.*, 2014
*Aedes aegypti*	Paraguay	1	patients in Reunion Island, 2005	06.21 and/or 06.115	7.5	10	n.d.	99 (89/90)	n.d.	Vega-Rua* et al.*, 2014
	Uruguay	1	patients in Reunion Island, 2005	06.21 and/or 06.115	7.5	10	n.d.	100 (60/60)	n.d.	Vega-Rua* et al.*, 2014
	Argentina	1	patients in Reunion Island, 2005	06.21 and/or 06.115	7.5	10	n.d.	99 (119/120)	n.d.	Vega-Rua* et al.*, 2014
	Bangkok, Thailand	2	patient in Thailand, 1962	15561	5.3	7	66 (20/30)	66 (20/30)	n.d.	Turell* et al.*, 1992
	Ho Chi Minh City, Vietnam	colony	patient in Reunion Island, 2005 (E1226A)	06.115	7	14	n.d.	66 (135/206)	n.d.	Vazeille* et al.*, 2007
			patient in Reunion Island, 2005 (E1226V)	06.21			n.d	97 (227/234)	n.d.	
			patient in Mayotte, 2006	06.111			n.d.	92 (126/137)	n.d.	
			patient in Democratic Republic of Congo, 2000	06.117			n.d	78 (108/138)	n.d.	
	Higgs variant, Rexville D	colony	clone derived from patient in France ex. Reunion Island, 2006 E1226A	LR2006-OPY1 E1A226	5	7	20 (not stated)	n.d.	n.d.	Tsetsarkin * et al.*, 2007 [9]
			clone derived from patient in France ex. Reunion Island, 2006 E1226V	LR2006-OPY1 E1V226			5 (not stated)	n.d.	n.d.	
			infectious clone from patient in West Africa, E1226A	37997 E1A226			20 (not stated)	n.d.	n.d.	
			infectious clone from patient in West Africa, E1226V	37997 E1V226			10 (not stated)	n.d.	n.d.	
			Aedes furcifer, Kadougou, Senegal, 1983	37997	8	14	100 (7/7)	63 (5/8)	n.d.	Vanlandingham * et al.*, 2005 [189]
	not stated	colony	patient in Africa, not stated	not stated	8.6-9.2	14	53 (24/45)	n.d.	44 (20/45)	Mangiafico, 1971 [190]
	Rockefeller	colony	patient in Thailand, 1962	15561	4.2-4.6	7	18 (9/50)	12 (6/50)	n.d.	Turell* et al.*, 1992
	Madeira Island, Spain	1	patient in Reunion Island, 2005 (E1226V)	06.21	7	14	n.d.	100 (27/27)	40 (4/10)	Vazeille* et al.*, 2012 [191]
*Aedes albopictus*	Queensland, Australia	7	patient in Melbourne ex. Mauritius, March 2006	not stated	3.9	14-15	92 (23/25)	92 (23/25)	32 (8/25)	van den Hurk* et al.*, 2010
	Torres Strait, Australia	7	patient in Melbourne ex. Mauritius, March 2006	not stated	8	14	80 (4/5)	80 (4/5)	60 (3/5)	Nicholson* et al.*, 2014 [192]
	Mauritius	1	patient in India, 1973	Barsi, P0-731460	5.8	8-9	32 (13/41)	n.d.	n.d.	Tesh* et al.*, 1976 [193]
			patient in Tanzania, 1953	Ross, S-27	6.8	8-9	19 (13/67)	n.d.	n.d.	Tesh* et al.*, 1976
	Mayotte	1	patient in Reunion Island, 2005 (E1226A)	6.115	7	14	n.d	25 (115/462)	n.d.	Vazeille* et al.*, 2007
		6	patient in Reunion Island, 2005 (E1226A)	6.115	7.5	14	n.d.	79 (45/57)	n.d.	Martin* et al.*, 2010 [94]
		1	patient in Reunion Island, 2005 (E1226V)	6.21	7	14	n.d	91 (296/325)	n.d.	Vazeille* et al.*, 2007
		6	patient in Reunion Island, 2005 (E1226V)	6.21	7.5	14	n.d.	99 (64/65)	n.d.	Martin* et al.*, 2010
		1	patient in Mayotte, 2006	06.111	7	14	n.d.	98 (48/49)	n.d.	Vazeille* et al.*, 2007
		1	patient in Democratic Republic of Congo, 2000	06.117	7	14	n.d	73 (41/56)	n.d.	Vazeille* et al.*, 2007
	Reunion Island	2	patient in Reunion Island, 2005 (E1226A)	6.115	7.5	14	n.d.	90 (26/29)	n.d.	Martin* et al.*, 2010
		1	patient in Reunion Island, 2005 (E1226A)	6.115	7	14	n.d.	25 (114/462)	n.d.	Vazeille* et al.*, 2007
		2	patient in Reunion Island, 2005 (E1226V)	6.21	7.5	14	n.d.	98 (55/56)	n.d.	Martin* et al.*, 2010
		1	patient in Reunion Island, 2005 (E1226V)	6.21	7	14	n.d	96 (391/409)	n.d.	Vazeille* et al.*, 2007
		2	patient in Mayotte, 2006	6.111	7	14	n.d.	97 (91/94)	n.d.	Vazeille* et al.*, 2007
		2	patient in Democratic Republic of Congo, 2000	6.117	7	14	n.d	80 (25/31)	n.d.	Vazeille* et al.*, 2007
	Madagascar	7	patient in Thailand, 1962	15561	5.3	7	95 (19/20)	35 (7/20)	n.d.	Turell* et al.*, 1992
		1	patient in India, 1973	Barsi, P0-731460	6.2	8–9	87 (33/38)	n.d.	n.d.	Tesh* et al.*, 1976
		1	patient in Tanzania, 1953	Ross, S-27	6.8	8–9	39 (19/49)	n.d.	n.d.	Tesh* et al.*, 1976
		2-5	patient in Reunion Island, 2006	not stated	7.5	14	n.d.	98 (497/503)	n.d.	Raharimalala* et al.*, 2012 [76]
*Aedes albopictus*	Cameroon	1	patient in Reunion Island, 2006	06.21	7	14	n.d.	85 (187/218)	n.d.	Paupy* et al.*, 2010
			patient in Reunion Island, 2005 (E1226V)	06.21	7	14	n.d	68 (41/60)	n.d.	Vazeille* et al.*, 2007
			patient in Mayotte, 2006	06.111	7	14	n.d.	44 (34/44)	n.d.	Vazeille* et al.*, 2007
			patient in Democratic Republic of Congo, 2000	06.117	7	14	n.d	56 (22/39)	n.d.	Vazeille* et al.*, 2007
			patient in Reunion Island, 2005 (E1226A)	06.115	7	14	n.d	12 (11/90)	n.d.	Vazeille* et al.*, 2007
	Virginia and Georgia, USA	1	mosquito pool, Comoros, 2005 strain COM125	COM125	4.9	7	73 (83/114)	n.d.	40 (33/83)	McTighe & Vaidyanathan, 2012
	Hawaii, USA	1	patient in Tanzania, 1953	Ross, S-27	7-7.5	8-9	69 (76/110)	n.d.	n.d.	Tesh* et al.*, 1976
		1	patient in India, 1973	Barsi, P0-731460	5.8	8-9	97 (120/124)	n.d.	n.d.	Tesh* et al.*, 1976
		colony	patient in Calcutta, India, 1963	63-266	8	14	100 (32/32)	n.d.	34 (8/22)	Shah* et al.*, 1964
		colony	patient in Thailand, 1962	15561	5.3	7	93 (28/30)	60 (18/30)	n.d.	Turell* et al.*, 1992
	Florida, USA	1	patient in France ex. Reunion Island, 2006	LR2006-OPY1	6.1	6	100 (22/22)	91 (20/22)	n.d.	Pesko* et al.*, 2009
			patients in Reunion Island, 2005	06.21 and/or 06.115	7.5	10	n.d.	83 (50/60)	n.d.	Vega-Rua* et al.*, 2014
		2	patient in Thailand, 1962	15561	5.3	7	97 (29/30)	37 (11/30)	n.d.	Turell* et al.*, 1992
	Missouri, USA	1	patients in Reunion Island, 2005	06.21 and/or 06.115	7.5	10	n.d.	90 (54/60)	n.d.	Vega-Rua* et al.*, 2014
*Aedes albopictus*	Texas, USA	colony	clone derived from patient in France ex. Reunion Island, 2006 E1226A	LR2006-OPY1 E1A226	5	7	31 (61/194)	30 (not stated)		Tsetsarkin* et al.*, 2007
			clone derived from patient in France ex. Reunion Island, 2006 E1226V	LR2006-OPY1 E1V226	5	7	90 (241/269)	65 (not stated)		Tsetsarkin* et al.*, 2007
			infectious clone from patient in West Africa, E1226A	37997 E1A226	5	7	37 (97/226)	n.d.	n.d.	Tsetsarkin* et al.*, 2007
			infectious clone from patient in West Africa, E1226V	37997 E1V226	5	7	92 (253/274)	n.d.	n.d.	Tsetsarkin* et al.*, 2007
		9-10	patient in Thailand, 1962	15561	5.3	7	75 (15/20)	35 (7/20)	n.d.	Turell* et al.*, 1992
	Louisiana, USA	4-5	patient in Thailand, 1962	15561	5.3	7	97 (29/30)	80 (24/30)	n.d.	Turell* et al.*, 1992
	Mexico	1	patients in Reunion Island, 2005	06.21 and/or 06.115	7.5	10	n.d.	70 (42/60)	n.d.	Vega-Rua* et al.*, 2014
	Panama	1	patients in Reunion Island, 2005	06.21 and/or 06.115	7.5	10	n.d.	95 (57/60)	n.d.	Vega-Rua* et al.*, 2014
			patient in New Caledonia, 2011	NC/2011-568	7.5	10	n.d.	97 (29/30)	n.d.	Vega-Rua* et al.*, 2014
	Brazil	6-7	patient in Thailand, 1962	15561	5.3	7	73 (22/30)	50 (15/30)	n.d.	Turell* et al.*, 1992
		1	patients in Reunion Island, 2005	06.21 and/or 06.115	7.5	10	n.d.	94 (301/320)	n.d.	Vega-Rua* et al.*, 2014
	Argentina	1	patients in Reunion Island, 2005	06.21 and/or 06.115	7.5	10	n.d.	63 (35/56)	n.d.	Vega-Rua* et al.*, 2014
			patient in New Caledonia, 2011	06.21 and/or 06.115	7.5	10	n.d.	93 (28/30)	n.d.	Vega-Rua* et al.*, 2014
	Israel	1	patient in Tanzania, 1953	Ross, S-27	7.2	8-9	30 (14/47)	n.d.	n.d.	Tesh* et al.*, 1976
			patient in India, 1973	Barsi, P0-731460	5.8	8-9	67 (37/55)	n.d.	n.d.	Tesh* et al.*, 1976
	Lebanon	1	patient in Reunion Island, 2005 (E1226V)	6.21	8	14	n.d.	29 (12/42)	n.d.	Haddad* et al.*, 2012 [194]
*Aedes albopictus*	Italy	0	patient in Reunion Island, 2005 (E1226V)	06.21	7	14	n.d.	83 (52/63)	n.d.	Talbalaghi* et al.*, 2010 [195]
	France	0	patient in Reunion Island, 2005 (E1226V)	6.21	7	14	n.d.	77.1 (27/35)	n.d.	Vazeille* et al.*, 2008 [196]
		13	patient in France, 2010 (E1226A)	1909	7.3	14	n.d.	96 (21/22)	14 (3/21)	Vega-Rua* et al.*, 2013 [36]
		13	patient in France, 2010 (E1226A)	1630	7.3	14	n.d.	90 (17/19)	12 (2/17)	Vega-Rua* et al.*, 2013
	Corsica, France	0	patient in Reunion Island, 2005 (E1226V)	6.21	7.5	14	n.d.	94 (377/401)	n.d.	Moutailler* et al.*, 2009 [197]
	Indonesia	1	patient in Tanzania, 1953	Ross, S-27	7.1	8–9	64 34/53)	n.d.	n.d.	Tesh* et al.*, 1976
	Philippines	1	patient in Tanzania, 1953	Ross, S-27	7.2	8–9	55 (21/38)	n.d.	n.d.	Tesh* et al.*, 1976
	India	1	patient in Tanzania, 1953	Ross, S-27	7–7.2	8–9	38 (30/79)	n.d.	n.d.	Tesh* et al.*, 1976
			patient in India, 1973	Barsi, P0-731460	5.7–5.9	8–9	71 (74/104)	n.d.	n.d.	Tesh* et al.*, 1976
	Vietnam	1	patient in Tanzania, 1953	Ross, S-27	7.7	8–9	44 (29/66)	n.d.	n.d.	Tesh* et al.*, 1976
			patient in India, 1978	Barsi, P0-731460	5.7	8–9	49 (37/94)	n.d.	n.d.	Tesh* et al.*, 1976
	Thailand	1	patient in Tanzania, 1953	Ross, S-27	7.4	8–9	38 (12/32)	n.d.	n.d.	Tesh* et al.*, 1976
			patient in India, 1973	Barsi, P0-731460	6	8–9	73 (24/33)	n.d.	n.d.	Tesh* et al.*, 1976
	Malaysia	1	patient in Tanzania, 1953	Ross, S-27	6.9	8–9	29 (15/51)	n.d.	n.d.	Tesh* et al.*, 1976
			patient in India, 1973	Barsi, P0-731460	6	8–9	42 (27/64)	n.d.	n.d.	Tesh* et al.*, 1976
	Taipei	1	patient in Tanzania, 1953	Ross, S-27	7	8–9	25 (12/48)	n.d.	n.d.	Tesh* et al.*, 1976
			patient in India, 1973	Barsi, P0-731460	5.8	8–9	28 (14/50)	n.d.	n.d.	Tesh* et al.*, 1976
	Taiwan	2	patient in Thailand, 1962	15561	5.3	7	90 (27/30)	20 (6/30)	n.d.	Turell* et al.*, 1992
	Okinawa, Japan	5	patient in Thailand, 1962	15561	5.3	7	72 (18/25)	28 (7/25)	n.d.	Turell* et al.*, 1992
	Tokyo, Japan	5	patient in Thailand, 1962	15561	4.2–4.6	7	50 (14/28)	14 (7/50	n.d.	Turell* et al.*, 1992
	Jakarta	1	patient in India, 1975	Barsi, P0-731460	5.8	8–9	70 (23/33)	n.d.	n.d.	Tesh* et al.*, 1976
	Philippines	1	patient in India, 1976	Barsi, P0-731460	6	8–9	87 (33/38)	n.d.	n.d.	Tesh* et al.*, 1976
	Sabah, Malaysia	5	patient in Thailand, 1962	15561	4.2–4.6	7	43 (15/35)	6 (2/43)	n.d.	Turell* et al.*, 1992
*Aedes albopictus*	Hanoi, Vietnam	3	patient in Reunion Island, 2005 (E1226A)	6.115	7	14	n.d	30 (16/54)	n.d.	Vazeille* et al.*, 2007
			patient in Reunion Island, 2005 (E1226V)	6.21	7	14	n.d	84 (105/126)	n.d.	Vazeille* et al.*, 2007
			patient in Mayotte, 2006	6.111	7	14	n.d.	84 (105/126)	n.d.	Vazeille* et al.*, 2007
			patient in Democratic Republic of Congo, 2000	6.117	7	14	n.d	47 (56/119)	n.d.	Vazeille* et al.*, 2007
	not stated	colony	patient in Africa, not stated	not stated	8.6–9.2	14	100 (50/50)	n.d.	80 (40/50)	Mangiafico, 1971
*Aedes antipodeus*	North Auckland, New Zealand	1	patient in India	91064A	7.8	21**	100 (15/15)	73 (11/15)	0 (0/15)	Kramer* et al.*, 2011 [198]
*Aedes caspius*	France	0	patient in Reunion Island, 2005 (E1226V)	06.21	7	14	n.d.	25 (4/16)	n.d.	Vazeille* et al.*, 2008
*Aedes detritus*	France	0	patient in Reunion Island, 2005 (E1226V)	06.21	7	14	n.d.	67.3 (33/49)	n.d.	Vazeille* et al.*, 2008
*Aedes fulgens*	South Africa	1	not stated	H817	5.7	9–12	88 (29/33)	n.d.	10 (3/29)	Jupp* et al.*, 1981 [199]
*Aedes furcifer*	South Africa	colony or 1-4	not stated	H817	5.7–6.9	8–29	71 (192/271)	n.d.	30 (8/27)	Jupp* et al.*, 1981
*Aedes hensilli*	Micronesia	12-15	Mosquito in Comoros, 2005	COM 125	5.7	8	63 (20/32)	80 (16/20)	n.d.	Ledermann* et al.*, 2014 [200]
*Aedes notoscriptus*	Auckland, New Zealand	1	patient in India	91064A	10.5	14*	36 (8/32)	75 (6/8)	0 (0/8)	Kramer* et al.*, 2011
*Aedes polynesiensis*	Samoa	colony	patient in Calcutta, India, 1963	63-266	8	14	40 (4/10)	n.d.	n.d.	Shah* et al.*, 1964
*Aedes togoi*	not stated	colony	patient in Africa, not stated	not stated	8.6–9.2	14	97 (42/43)	n.d.	12 (5/43)	Mangiafico, 1971
*Aedes triseriatus*	not stated	colony	patient in Africa, not stated	not stated	8.6–9.2	14	100 (50/50)	n.d.	84 (42/50)	Mangiafico, 1971
*Aedes vexans*	Italy	0	patient in Reunion Island, 2005 (E1226V)	06.21	7	14	n.d.	8 (2/26)	n.d.	Talbalaghi* et al.*, 2010
	France		patient in Reunion Island, 2005 (E1226V)	06.21	7	14	n.d.	0 (0/13)	n.d.	Vazeille* et al.*, 2008
*Aedes vittatus*	Senegal	1	mosquitoes, bats or humans in Senegal, 1962, '79 and '05	ArD30237, CS13-288 or HD 180738	6	10	89 (41/46)	54 (22/41)	18 (4/22)	Diagne* et al.*, 2014 [201]
					6-7	10	19 (19/98)	37 (7/19)	43 (3/7)	Diagne* et al.*, 2014
*Anopheles gambiae*	G3	colony	*Aedes furcifer*, Kadoug ou, Senegal, 1983	37997	8	14	0 (0/8)	0 (0/8)	n.d.	Vanlandingham* et al.*, 2005
*Anopheles maculipennis*	Italy	0	patient in Reunion Island, 2005 (E1226V)	06.21	7	14	n.d.	0 (0/10)	n.d.	Talbalaghi* et al.*, 2010
*Culex fatigans*	Philippines	colony	patient in Calcutta, India, 1963	63-266	8	14	0 (0/10)	n.d.	n.d.	Shah* et al.*, 1964
*Culex horridus*	South Africa	1	not stated	H817	4.6–5.4	14–25	6 (1/17)	n.d.	n.d.	Jupp* et al.*, 1981
*Culex pipiens*	Italy	0	patient in Reunion Island, 2005 (E1226V)	06.21	7	14	n.d.	0 (0/45)	n.d.	Talbalaghi* et al.*, 2010
	France		patient in Reunion Island, 2005 (E1226V)	06.21	7	14	n.d.	0 (0/11)	n.d.	Vazeille* et al.*, 2008
*Culex quinquefasciatus*	Zimbabwe	1	not stated	H817	5.3	20–22	0 (0/19)	n.d.	0 (0/19)	Jupp* et al.*, 1981
*Eretmapodites chrysogaster*	not stated	colony	patient in Africa, not stated	not stated	8.6–9.2	14	80 (40/50)	n.d.	36 (18/50)	Mangiafico, 1971
*Mansonia africana*	Mozambique	0	not stated	H817	4.7–5.2	8–15	34 (23/67)	n.d.	n.d.	Jupp* et al.*, 1981
*Opifex fuscus*	Wellington, New Zealand	1	patient in India	91064A	6.2	11*	98 (46/47)	100 (46/46)	100 (46/46)	Kramer* et al.*, 2011
*Ornithodoros savignyi*	South Africa	0	not stated	H817	6.6	50-61	0 (0/11)	n.d.	n.d.	Jupp* et al.*, 1981

## 6. Conclusions

Chikungunya virus has caused explosive outbreaks of severe, debilitating and often chronic arthralgia since it emerged in the 1950s and later in 2004 from the enzootic ECSA lineage in Africa. The large populations of susceptible humans in many naïve regions and thriving populations of the two urban vectors, *A. aegypti* and *A. albopictus*, will probably facilitate endemicity throughout most regions of the tropics and subtropics for the foreseeable future, although the burden of disease is typically difficult to estimate because CHIKV and DENV infections are difficult to distinguish clinically. Further adaptation of CHIKV to these urban vectors is also suggested by recent findings [13]. Future emergences from enzootic African cycles will also remain a risk that is increasing with more and more air travel and international commerce. Although novel strategies to control vector populations and reduce transmission are in early stages of field testing, the deployment of one of several highly promising human vaccines probably offers the best hope for making a major impact in restricting CHIKV circulation and preventing human disease [184].
